# A rare case of primary chondrosarcoma arising from the sternum: A case report

**DOI:** 10.3892/ol.2014.2453

**Published:** 2014-08-18

**Authors:** BIN HE, YUEHAN HUANG, PENG LI, XIAOHE YE, FUQIANG LIN, LIDONG HUANG, SHENGQIANG GAO, HONGQI SHI, YUNFENG SHAN

**Affiliations:** 1 Department of Hepatobiliary Surgery, The First Affiliated Hospital of Wenzhou Medical University, Wenzhou, Zhejiang 325000, P.R. China; 2 Department of Pathology, The First Affiliated Hospital of Wenzhou Medical University, Wenzhou, Zhejiang 325000, P.R. China; 3 Department of Radiology, The First Affiliated Hospital of Wenzhou Medical University, Wenzhou, Zhejiang 325000, P.R. China; 4 Department of Cardiac and Thoracic Surgery, The First Affiliated Hospital of Wenzhou Medical University, Wenzhou, Zhejiang 325000, P.R. China

**Keywords:** positron emission tomography-computed tomography, chondrosarcoma, sternum, titanium mesh

## Abstract

The current study reports a case of an extremely rare tumor that presented in an uncommon location, which was successfully treated via radical resection and reconstruction. A 37-year-old female, with no notable medical history, with the exception of a cesarean delivery, was admitted to The First Affiliated Hospital of Wenzhou Medical University (Wenzhou, China) due to pain and a lump of the anterior chest wall. The mass was identified on the manubrium sterni and was not tender on palpation. A chest computed tomography (CT) scan reconstruction identified the abnormal mass on the manubrium sterni (size, 5×4 cm in diameter) and positron emission tomography-CT interpretation strongly indicated a type of well-differentiated malignant tumor, such as a giant cell tumor. An aspiration needle biopsy was not conducted, however, the patient underwent tumor radical resection and sternal reconstruction using steel wire and titanium mesh. Histopathological examination of the surgical specimen determined the diagnosis of chondrosarcoma. A postoperative chest X-ray revealed that the sternal defect had repaired well, therefore, this procedure may be highly beneficial in future for repairing defects in the sternum.

## Introduction

Primary sternal tumors are a rare type of bone and soft tissue tumor, with chondrosarcoma being the most common. Generally, the precise evaluation of such tumors requires a thorough radiological examination since the clinically appreciable mass usually involves the sternum (1). However, sternal chondrosarcomas are resistant to chemotherapy and radiation and thus, wide resection is the only curative option (2). Sternal tumors have been considered a significant challenge in surgery due to the difficulties in full-thickness resectioning without compromising the stability and reconstruction of the thoracic wall (1). Due to improvements in surgical technique, it is now possible to perform total sternectomies (1). Adequate surgical excision and reconstruction may offer a definitive cure; however, the surgical procedure may be difficult to perform due to the local aggressiveness of these tumors and their high recurrence rate and thus, reconstruction remains controversial (3). In addition, chest wall stability following wide sternectomy may be achieved by using various prosthetic materials, such as Marlex mesh or polytetrafluoroethylene patches (4). The aims of the current study were to report a rare case of chondrosarcoma of the manubrium sterni, which was successfully treated via radical resection and reconstruction with titanium mesh and steel wire and to evaluate the use of such techniques. Written informed consent was obtained from the patient.

## Case report

**Figure 1:**
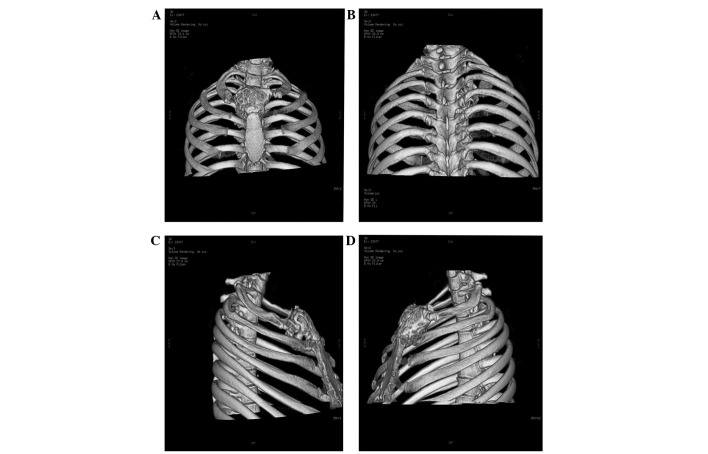
Preoperative chest computed tomographic reconstruction revealed a mass located in the manubrium sterni. (A) Anterior aspect, (B) posterior aspect, (C) left lateral aspect and (D) right lateral aspect.

**Figure 2:**
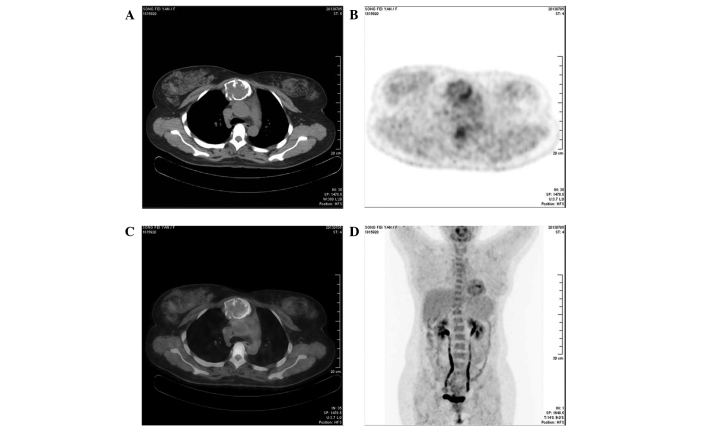
(A and C) On the basis of the anatomic and metabolic findings, the positron emission tomography-computed tomography indicated a well differentiated malignant tumor. (B and D) Sequential transaxial views of the chest indicated that the manubrium sterni mass was slightly 18F-fluorodeoxyglucose-avid with a maximum standardized uptake value of 3.7.

In July 2013, a 37-year-old previously healthy female, who had previously undergone one cesarean delivery, presented at The First Affiliated Hospital of Wenzhou Medical University (Wenzhou, China) with a lump and pain of the anterior chest wall. On physical examination, a palpable mass was identified on the manubrium sterni, which was not tender on palpation. Laboratory tests revealed no abnormalities; however, a chest computed tomography (CT) scan reconstruction demonstrated an expansive localized, hypodense, round mass on the manubrium sterni (diameter, ~5.1×4.2 cm; Fig. 1). The CT indicated that the mass may be a giant cell tumor. Positron emission tomography (PET)-CT revealed an expanding mass with an irregular shape, arising from the manubrium sterni, with a maximum diameter of 6 cm and a maximum standardized uptake value of 3.7 (Fig. 2). The mass was marginally ^18^F-fluorodeoxyglucose (FDG)-avid, however, there was no evidence that the distant metastases were FDG-avid. On the basis of the anatomic and metabolic findings, the PET-CT interpretation strongly indicated a type of well-differentiated malignant tumor, such as a giant cell tumor.

With a clear resection margin, a radical resection and reconstruction using steel wire and titanium mesh (M3 system/model; OsteoMed Co., Addison, TX, USA) was performed. Radical resection of the right and left sternoclavicular joints, the second rib and the entire upper section of the sternum body was performed, and a drainage tube was placed in the mediastinum. Following surgery, the patient received antibiotics and expectorant treatment, along with parenteral nutrition. The pain subsequently diminished and became tolerable. On postoperative day five, the removal of the mediastinal drainage tube was required; the patient recovered well following surgery and was discharged on postoperative day eight. The overall duration of the hospital stay was 16 days. Follow-up CT scans were performed every three months to monitor tumor recurrence. After 12 months of follow-up no tumor reccurence was identified.

**Figure 3:**
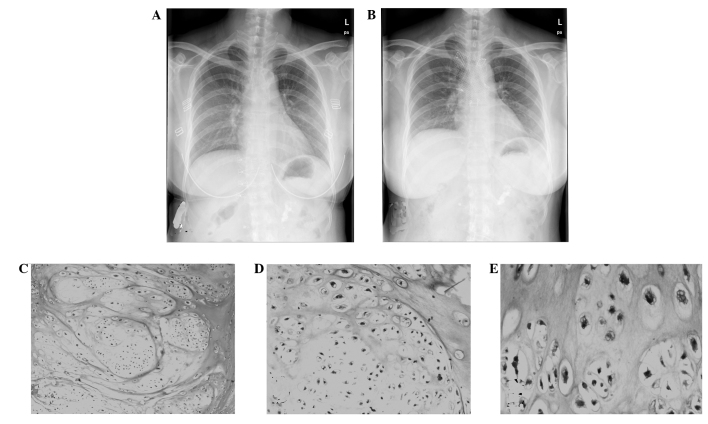
(A) Chest X-ray of a 37-year-old female who presented with a mass and pain in the anterior chest wall, however, had not exhibited any indication of a sternum mass prior to surgery. (B) Chest X-ray one month following radical resection of the tumor and chest wall reconstruction using titanium mesh revealed the sternal defect had repaired well. (C–E) Histopathological examination of the surgical specimen (stain, hematoxylin and eosin) indicated a diagnosis of chondrosarcoma. The specimen was predominantly composed of hyaline cartilage cells and a chondromyxoid cartilage matrix, as well as atypical cells, including binucleated forms. Magnification; (C) ×40; (D) ×100; and (E) ×200.

Histological analysis of a surgical specimen revealed well-differentiated malignant cartilaginous cells, consistent with a grade I (considered to be low grade) chondrosarcoma (Fig. 3). The tumor cells were located within the medullary bone, resulting in bone expansion and destruction of the bone cortex. The chondrosarcoma was predominantly composed of hyaline cartilage cells and exhibited a chondromyxoid cartilage matrix, as well as atypical cells, including binucleated cells.

## Discussion

Primary sternal tumors are uncommon and account for only ~1% of primary bone neoplasms, worldwide (1,5). Chondrosarcoma, a type of malignant cartilage-forming tumor, is considered to be the most common primary malignancy of the anterior thoracic wall. However, chondrosarcomas remain a rare type of lesion in the sternum and the majority of neoplasms are secondary sternal tumors, which arise from metastases originating in areas, such as the thyroid, breasts and lungs (6,7).

In cases of chondrosarcoma, the CT scan usually indicates a hypodense, rounded mass that is located in the sternum; the bone is expansive and the cortex is thin and damaged. PET-CT may occasionally distinguish benign from malignant bone neoplasms, however, certain well-differentiated tumors may be only marginally FDG-avid on PET-CT imaging, therefore, it can be challenging to differentiate the tumor types. Although the histopathological findings of the excised mass are the only true determinant of the nature of the cancer, PET-CT remains a valuable and meaningful measure for assessing the nature and extent of the tumor.

Histopathologically, chondrosarcoma exhibit a distinctive architecture, with high numbers of hyaline cartilage cells and the presence of a chondromyxoid cartilage matrix. The atypical cells are comprised of oval or round nuclei and a deeply eosinophilic cytoplasm (8).

Bone autografts and allografts from the ribs or iliac bone have been reported as an alternative to synthetic materials for use when rebuilding chest wall defects (9). However, omental flaps and titanium plates offer increased stability and safety for reconstruction following extensive sternocostal resection (10). Due to its biocompatibility and flexibility, titanium mesh may also be an effective material for reconstructing defects without limitations (5). It has been associated with minimal trauma, fewer infections and reduced postoperative pain. Furthermore, following surgery, patients recover well, which results in a short hospital stay.

In conclusion, the current study reports a rare case of primary chondrosarcoma of the sternum, which was successfully treated by radical resection and reconstruction using steel wire and titanium mesh. Therefore, in future cases, the use of titanium mesh and steel wire may be considered as highly beneficial materials for use during the reconstruction of defects of the sternum.

## References

[1] Lequaglie C, Massone PB, Giudice G, Conti B (2002). Gold standard for sternectomies and plastic reconstructions after resections for primary or secondary sternal neoplasms. Ann Surg Oncol.

[2] Kużdżał J, Warmus J, Grochowski Z, Gądek A (2014). Reconstruction of the sternal manubrium. J Thorac Cardiovasc Surg.

[3] Chapelier AR, Missana MC, Couturaud B (2004). Sternal resection and reconstruction for primary malignant tumors. Ann Thorac Surg.

[4] McCormack P, Bains MS, Beattie EJ, Martini N (1981). New trends in skeletal reconstruction after resection of chest wall tumors. Ann Thorac Surg.

[5] Liu ZC, Zhao H (2010). Titanium internal fixation system used for sternum reconstruction after resection of chondrosarcoma. Chin Med J (Engl).

[6] Alpert JB, Nonaka D, Chachoua A, Pass HI, Ko JP (2011). Increasing dyspnea due to an anterior mediastinal mass. Chest.

[7] Stanić V, Vulović T, Novaković M (2008). Radical resection of giant chondrosarcoma of the anterior chest wall. Vojnosanit Pregl.

[8] Waisberg DR, Abrão FC, Fernandez A, Terra RM, Pêgo-Fernandes PM, Jatene FB (2011). Surgically-challenging chondrosarcomas of the chest wall: five-year follow-up at a single institution. Clinics (Sao Paulo).

[9] Marulli G, Hamad AM, Cogliati E, Breda C, Zuin A, Rea F (2010). Allograft sternochondral replacement after resection of large sternal chondrosarcoma. J Thorac Cardiovasc Surg.

[10] Rocco G, Fazioli F, La Manna C (2010). Omental flap and titanium plates provide structural stability and protection of the mediastinum after extensive sternocostal resection. Ann Thorac Surg.

